# Engagement in Culturally and Regionally Relevant Physical Activity (Hula, Outrigger Paddling, Spearfishing and Surfing) in the State of Hawaiʻi over the Lifecourse: A cross-sectional analysis

**DOI:** 10.21203/rs.3.rs-8225534/v1

**Published:** 2025-12-04

**Authors:** Catherine M. Pirkle, Mika D. Thompson, Rebekah Rodericks, Simone M. Schmid, Lance Kaeo Ching, Mele A. Look, Yan Yan Wu, Michael M. Phillips, Tetine Lynn Sentell

**Affiliations:** University of Hawaiʻi at Mānoa; University of Hawaiʻi at Mānoa; University of Hawaiʻi at Mānoa; University of Hawaiʻi at Mānoa; Hawaii State Department of Health; University of Hawaiʻi at Mānoa; University of Hawaiʻi at Mānoa; University of Hawaiʻi at Mānoa; University of Hawaiʻi at Mānoa

**Keywords:** Pacific, Physical Activity, Lifecourse, Hula, Outrigger Paddling, Spearfishing, Surfing

## Abstract

**Introduction:**

Culturally and regionally relevant physical activities (CRRPA) show promise for disease prevention and strengths-based public health promotion. Understanding engagement across the lifecourse can support effective public health policy, research, and practice. This study examined: (1) factors associated with CRRPA participation, including gender, race/ethnicity, and chronic disease; (2) contexts of practice, as well as the frequency and duration of engagement; and, (3) lifetime participation in four CRRPA in Hawaiʻi (Hula, outrigger canoe paddling, spearfishing, and surfing).

**Methods:**

A cross-sectional analysis was conducted among 1044 English-speaking adults in Hawaiʻi, recruited through an online panel or by random-digit dialing to complete a survey examining CRRPA engagement in the state. Native Hawaiians and residents of rural islands (excluding Oʻahu) were oversampled for representation. Lifetime participation in each CRRPA was self-reported and dichotomized. Engagement was characterized by sociodemographic and health-related variables. Frequency, context, and duration were summarized for the total sample and for Native Hawaiians (n = 425). Engagement patterns over the lifecourse are also presented.

**Results:**

Overall, 69.3% of respondents reported lifetime participation in at least one CRRPA. In the full sample, 44.9% engaged in Hula, 41.4% in surfing, 34.9% in spearfishing, and 33.9% in outrigger canoe paddling. Among those reporting having engaged in at least one CRRPA (n = 724), percentages were 64.8% (hula), 59.7% (surfing), 49.7% (spearfishing), and 48.9% (paddling). Most participated in multiple CRRPA, and 12.4% engaged in all four at some point in their lives. Among Native Hawaiians, family-based participation was common, especially for spearfishing and surfing. The average session duration for all activities was two hours or more, with more frequent engagement reported among Native Hawaiians. Engagement was highest during earlier life stages and declined with age. Trends varied notably by generation, with younger generations engaging more than older ones. Lifetime patterns corresponded with important historical events that popularized activities such as Hula and surfing.

**Conclusions:**

Engagement in CRRPA is widespread across Hawaiʻi. Trends varied by demographic and generational factors, underscoring opportunities to advance strengths-based public health promotion, planning, and policy for health equity.

## Background

Insufficient physical activity (PA), estimated at 31% among adults worldwide (Strain et al., 2024), contributes significantly to global morbidity and mortality. The World Health Organization defines insufficient activity for adults as not doing at least 150 minutes of moderate-intensity activity per week, 75 minutes of vigorous-intensity, or a combination of these (Strain et al., 2024). Physical inactivity is a modifiable risk factor for a wide array of chronic health conditions, including stroke, hypertension, type 2 diabetes, coronary heart disease, several types of cancer, dementia, depression, and all-cause mortality (Katzmarzyk et al., 2022). The economic burden of physical inactivity is staggering. If PA levels are not increased, the total cost of physical inactivity globally is estimated at INT$520 billion between 2020–2030 (Santos et al., 2023). Increasing PA engagement is therefore essential to reduce chronic diseases, premature mortality, and healthcare costs, requiring innovative and sustainable PA promotion strategies and interventions.

For PA strategies and interventions to be effective and sustainable, they need to be meaningful and enjoyable to the people who engage in them (Lakicevic et al., 2020; Rodrigues et al., 2020). Research on PA from around the globe documents large variation in engagement, with some groups less likely to be sufficiently active. For example, insufficient PA varies considerably by region, sex, and age (Strain et al., 2024). Women are consistently less physically active than men and physical inactivity increases for both sexes after 60 years of age (Strain et al., 2024). Physical inactivity tends to be associated with factors correlated with social and health disparities. In the United States, physical inactivity is more common among those with lower incomes and educational levels (Xu et al., 2023). It is also more prevalent in communities experiencing the lasting effects of colonialism and historical marginalization (Bantham et al., 2021; Coble & Rhodes, 2006; Gidgup et al., 2022). For groups that are more physically inactive, health promotion activities should identify, promote, and assure accessibility to activities that resonate with these groups specifically.

In the State of Hawaiʻi, over 75% of adults experience insufficient PA (Hawaiʻi Department of Health, n.d.). Native Hawaiians (NH) are less likely to be sufficiently active. Statewide, 8.6% fewer NH meet aerobic PA guidelines compared to Whites (Hawaiʻi Health Matters, 2023b) and 9.4% fewer NH engage in monthly leisure-time PA compared to Whites (Hawaiʻi Health Matters, 2023a). While NH are among the fastest growing group in the United States (Ghosh, 2003), they also have some of the highest rates of chronic conditions in the country (King et al., 2012; Mau et al., 2009), with insufficient PA likely contributing to these conditions (Moy et al., 2010). The elevated prevalence of chronic conditions in NH contributes to the 4.4 year gap in life-expectancy between NH and Whites in Hawaiʻi (Wu et al., 2025).

Recent research in Hawaiʻi has revealed high engagement in several PAs of cultural relevance to NH and Pacific Islanders (PI), while also noting that participation in these culturally and regionally relevant PA may be undercounted in public health surveillance systems (Hansen et al., 2025; Sentell, 2023). In the State of Hawaiʻi, 25.5% of adults reported having engaged in spearfishing, 24.5% in Hula, and 19.8% in paddling at some point in their lifetime. While there was some variation, especially for spearfishing, overall engagement in these activities was robust across age groups, education, sex, and income levels. Engagement was even higher among NH (48.8% Hula, 41.5% paddling, 42.6% spearfishing). These findings underscore the need to better understand engagement in culturally grounded activities - its timing, variation across the lifespan, and associations with demographic and health factors. Given the enduring health impacts of colonization and historical trauma in Hawaiʻi (Blaisdell, 1993; Greywolf et al., 2025; Niheu et al., 2007), understanding NH engagement in these practices is essential for developing public health programs and policies aligned with community values, interests, and history.

Culturally and regionally relevant PA (CRRPA), defined as PA based on a population’s cultural customs, can support engagement in groups disproportionately at risk of being inactive, with proven effectiveness in disease prevention and management, as well as promise in public health promotion building from communities’ interests, strengths, and values (Hansen et al., 2025; Kaholokula et al., 2017; Look et al., 2012; Sentell, 2023; Van Duyn et al., 2007). A robust, interdisciplinary literature on this topic confirms that culturally relevant PAs are physically demanding, meaningful, and engaging forms of PA with health benefits that include chronic disease prevention, increased PA, and also address other areas of mental and spiritual wellbeing, as well as belongingness (Heil et al., 2019; Kaholokula et al., 2017; Look et al., 2023; Tang et al., 2022). For instance, participants of weekly Hula classes significantly lowered their blood pressure compared to those who did not attend the Hula classes (Kaholokula et al., 2021). As cultural practices, these activities can build strong relationships not only between participants, but also within families and communities. Furthermore, the shared values and ancestral knowledge inherent in these culturally-based PAs, especially those that align with cultural expectations, promote enduring engagement and ultimately, wellbeing (Kaholokula et al., 2018).

From a research and public health perspective, foundational questions remain about how to promote, scale, and sustain CRRPA at a population level. Little is known about who participates, the extent and frequency of engagement, when participation occurs or declines across the lifecourse, and the social contexts in which these activities take place. Thus, this study sought to: (1) explore factors associated with engagement in CRRPA over the lifecourse, including gender, race/ethnicity, and chronic disease; (2) ascertain the contexts of practice, as well as the frequency and duration of engagement; and, (3) illuminate trajectories in lifetime participation in four CRRPA for the state of Hawaiʻi (Hula, outrigger canoe paddling, spearfishing, and surfing).

### Types of CRRPA Practices in Hawaiʻi

Hula, is the indigenous dance of Native Hawaiians; its ancient origins included both sacred and secular purposes, and maintains broad popularity across Hawaiʻi and globally. It is an art form as well as a moderate-to-vigorous PA (Usagawa et al., 2014; Zhu et al., 2018) and functions as a form of creative expression and cultural identity, as well as maintains its traditional purpose of preserving history and stories (Kaeppler, 2004; Look et al., 2023). Hula is choreographed with specific controlled rhythmic movements that enhance or allude to the meaning or poetry of the accompanying songs or chants. Hula training typically occurs at hālau Hula (traditional Hawaiian dance and creative arts academy), where all genders, ethnicities, and dancers from children to elders receive cultural training and dance instruction (Galla et al., 2015; Look et al., 2023).

Traditional Pacific Island outrigger canoe paddling, referred to as paddling in this paper, can be in one, two, or six person canoes. The Hawaiian-style outrigger canoe, or *waʻa*, has a stabilizing outrigger float that is attached to the canoe hull by two perpendicularly orient struts (Haley & Nichols, 2009). Paddling is the official team sport of Hawaiʻi (Hawaiʻi State Capitol, 1986) and canoeing in general is considered a moderate-to-vigorous PA (Herrmann et al., 2024). Outrigger canoe paddling can be incredibly physically demanding, especially when training for competitive racing (Haley & Nichols, 2009; Sealey et al., 2011).

Spearfishing is an active form of fishing that involves the use of a specialized spear or harpoon to catch fish underwater (Sbragaglia et al., 2023). There are different approaches to spearfishing. One method is free diving, which relies on breath-holding and diving without an additional breathing apparatus, demanding considerable skill in breath control and swimming. However, spearfishing is also done with snorkeling gear or even SCUBA (Sbragaglia et al., 2023). Physical, psychological, and emotional wellbeing benefits have been linked to spearfishing, often associated with nature connectedness, as well as food acquisition, especially of high-quality fish (Sbragaglia et al., 2023; Young et al., 2016). Native Hawaiian expertise and cultural traditions related to spearfishing or oʻiʻa are well documented by historians, and many rites, traditions and practices recorded nearly 200 years ago continue to this day (Kahāʻulelio, 2006; Kamakau, 1976).

Surfing, the elevated form of wave-riding upright on a board originated with Native Hawaiians and has been extensively documented since first Western contact in the 18th Century (Clark, 2011). Hawaiians called this form of surfing heʻe nalu or “wave-sliding” which indicates the sport’s objective is not merely catching a wave but skillfully riding it. Hawaiian oral history dated around the 15th Century richly describes chiefs and deities surfing and competing in the same manner as surfers do today (Hoʻoulumāhiehie, 2006; Swanson, 2008). Hawaiian Olympian Duke Kahanamoku, in the early 20th Century, is credited with igniting the sport’s worldwide popularity, with now an estimated 50 million surfers globally, and recently established as an official Olympic sport (Clark, 2011; Rice, 2021). Surfing has been linked to mental and physical health benefits, greater community cohesion, and stronger intra-family relationships (Manero et al., 2024; Sentell et al., 2024). Despite this evidence, surfing is understudied in the context of health promotion (Rice, 2021).

It is noteworthy that several of these practices have distinct historical trajectories in Hawaiʻi. For example, Hula was suppressed during the early missionary period, then underwent a significant revival during the reign of King Kalākaua in the late 1800s. Surfing was similarly discouraged by missionaries in Hawaiʻi. The suppression and subsequent resurgence of activities such as Hula and surfing reflects both the enduring impacts of colonization and the resilience of Native Hawaiian communities in reclaiming cultural identity and wellbeing.

## Methods

### Study design and setting

A cross-sectional survey was conducted among Hawaiʻi residents recruited from an existing research panel managed by the market research and consulting firm, SMS Hawaiʻi. Survey fielding occurred from mid-June 2022 to mid-August 2022, with a target sample size of 1000 respondents.

### Sampling approach

SMS Hawaiʻi used LUCID, a panel aggregator that leverages different panel companies to get as wide a sample of Hawaiʻi residents as possible. Eligibility criteria included age (18–99) and a Hawaiʻi zip code. Online surveys were completed by 943 respondents recruited through the panel aggregator.

To enhance coverage and representation of individuals with limited internet access, particularly older adults, Random digit dialing and computer-assisted telephone interviewers were also used to meet the sampling target. For the phone sample, SMS Hawaiʻi used a random generator for both landline and cellphone numbers by island; 15,827 numbers were dialed and 150 surveys completed (140 after data cleaning). A sample with data from 1083 respondents was sent to the study authors. After data cleaning and management, the resulting sample was 1066. For analyses, an additional 22 individuals were excluded due to no response to the items on CRRPA for a final analytic sample of 1044.

### Participants

All adult Hawaiʻi residents could participate in the survey. An oversampling quota of 400 was requested for NH and other Pacific Islanders. This quota was achieved for NH, but not Pacific Islanders. These are island and racial/ethnic groups for whom CRRPA are particularly relevant (Hansen et al., 2025; Look et al., 2023; Sentell, 2023), but also tend to be underrepresented in survey research (Lee et al., 2022). 430 NH respondents completed the survey (425 in the analytic sample). Oversampling was also conducted for residents of Maui, Kauaʻi, and Hawaiʻi counties, which are much less populous than Honolulu County (island of Oʻahu) and primarily rural. Previous research has demonstrated significantly higher engagement in CRRPA among those residing in these counties than Honolulu (Hansen et al., 2025; Sentell, 2023).

### Outcomes

Lifetime engagement: Following previous research (Hansen et al., 2025; Sentell, 2023), respondents were asked “During your lifetime, how much have you participated in: 1) Hula, including during school, with friends and family, or in a hālau (school)?, 2) outrigger canoe paddling, including during school, with friends and family, or as part of a club?, 3) spearfishing, including free diving, scuba diving, reef walking, or torching for fish or tako/squid?, and 4) surfing, including during school, with friends and family, or as part of a club?”. Response choices were never, almost never, sometimes, often, very often, don’t know/not sure, and refused. Reports of “don’t know/not sure” or “refused” (n = 22) were treated as missing and excluded them from analyses.

Lifetime engagement was considered for each activity as a dichotomous variable (yes = sometimes, often, or very often vs no = never or almost never) based on previous research (Hansen et al., 2025; Sentell, 2023). A dichotomous yes/no variable was also constructed to capture activity in any of the four CRRPA (“any activity”). Additionally, we considered the overlap between each of the four activities among individuals, categorizing people into engagement in none, one, two, three, or four of the activities.

For those who reported having participated at least sometimes during their lifetime, additional questions were asked about this engagement:

1. Age of Participation over Lifetime: For all activities, timing of engagement across the lifecourse was assessed using the question “over your lifetime, which ages have you been involved in [each activity]?”. Response choices included: under 18 years old, 18–24 years old, 25–34 years old, 35–44 years old, 45–54 years old, 55–64 years old, 65–74 years old, and 75 or more years old. More than one age group could be selected. Age-of-participation responses were constrained by the respondent’s current age; participants could not report engagement in age ranges beyond their actual age.
2. Context of Activity: Hula and paddling respondents were asked: “where did you regularly participate?”: at school, with family, with friends, in hālau Hula/club, church, and/or other. For surfing and spearfishing, respondents were asked “when you regularly participated, were you”: alone, with family, with friends, with a club, church group, or other.
3. Frequency: respondents were asked: “during a typical month, how many times on average did you participate in [each activity]?”.
4. Duration: respondents were asked: “On the days you participated in [each activity], how long did you typically participate?”.

### Covariates

Demographics: “Race and ethnicity” categories followed Hawaiʻi State Department of Health standards: Native Hawaiians/Part NH (“NH sample”), Other Pacific Islanders, Filipino, Japanese, Chinese, White, and Other race/ethnicity. In accordance with standard practice in Hawaiʻi, if an individual selected more than one race/ethnicity and one of those included NH, they were categorized as NH. Additional covariates included age group (18–24, 25–34, 35–44, 45–54, 55–64, or ≥ 65 y), sex (female or male), location of residence (Oʻahu or neighbor islands), education (less than high school graduate; high school/some college; college degree), and annual household income (<$15,000, $15,000-$24,999, $25,000-$49,999, $50,000-$74,999, $75,000-$124,999, ≥$125,000).

Health status: Respondents were asked a number of health related questions including chronic conditions in the past five years (diabetes [yes/no], major depression [yes/no], and hypertension [yes/no]); self-rated health (excellent, very good, good, fair, or poor); as well as on mobility disability based on difficulty climbing steps or walking (any difficulty/no difficulty).

### Statistical Analyses

Descriptive statistics (counts and percentages) were calculated for lifetime engagement in each CRRPA, and overlap between the four activities. Additional descriptive statistics were calculated for the contexts of engagement and frequency (counts and percentages) and duration (mean and standard deviation). All analyses were unweighted.

For lifetime engagement, participants were limited to responding within categories that reflected their current and younger age group categories. Therefore, engagement by age was analyzed only for the under 18 and 18–24 age categories, which included the full sample. Respondents reported whether they had engaged in each activity during predefined age ranges (e.g., < 18, 18–24, 25–29, etc.). Using each respondent’s current age group, we aligned these retrospective age-of-engagement reports with the approximate calendar periods in which the reported engagement would have occurred. This allowed us to assign each endorsed age range to a corresponding decadal period (e.g., 1940s, 1950s) for descriptive plotting.

Given the cultural significance of CRRPA for NHs, analyses were repeated for the NH-only analytic sample (n = 425). Analyses were conducted using R version 4.4.0 (R Foundation for Statistical Computing).

## Results

### Participation

Over their lifetime, 69.3% of respondents participated in at least one of the four CRRPAs, with lifetime engagement at 44.9% for Hula, 41.4% for surfing, 34.5% for surfing, and 33.9% for paddling. Descriptive information about the sample and those participating in each activity for the overall sample can be found in Table 1. Respondents were 64.2% female and 40.7% Native Hawaiian, 23.2% White, 13.6% Japanese, 7.6% Filipino, 3.4% other race/ethnicity, 4.2% Chinese, 3.2% Other Pacific Islander, 2.4% Latino, and 1.8% Other Asian.

Similar proportions of men and women (67.9% and 70.2%, respectively) reported engaging in any of the four CRRPA activities during their lifetimes. Activity types differed by sex. Among women, Hula was the most common activity (55.5%), followed by surfing (36.7%), paddling (34.5%), and spearfishing (27.2%). Among men, the most common activity was surfing (49.7%) followed closely by spearfishing (47.6%). A third of men (32.9%) reported engaging in paddling during their lifetimes and a quarter (25.9%) in Hula (Table 1).

Across all race/ethnicity groups, except Chinese (43.2%), lifetime engagement in any activity was higher than 50%. Among NH, reported engagement in any activity was 86.4%. The next highest engagement was among the group categorized as “other race/ethnicity” (77.1%), followed by PI (72.7%), Latino (68.0%), Filipino (59.5%), and Japanese (57.0%). It should be noted that only 35 respondents fell into the category of “other race/ethnicity” and 33 into PI. Among NH, for specific activities, Hula (64.7%) was the most commonly reported activity, followed by surfing (53.7%), paddling (49.4%), and spearfishing (48.2%) (Table 1).

There was a reverse gradient in lifetime engagement in these activities by educational level; engagement was lowest among college graduates and highest among those who did not graduate high (secondary) school. This pattern was consistent across all activities, except paddling. Paddling engagement was lowest for college graduates, but similar across the other three education categories. There was no discernable pattern by household income. By county of residence, lifetime engagement was highest in Hawaiʻi county (76.2%), followed by Honolulu (68.8%), Maui (67.4%), and Kauaʻi (63.5%) counties (Table 1).

Across all activities, lifetime engagement was highest for those reporting excellent self-rated health. For paddling, spearfishing, surfing, and any activity, engagement was lowest for those reporting poor self-reported health. For Hula, lifetime engagement was lowest among those reporting good health (41.3%); among those reporting excellent health, 53.1% reported lifetime engagement (Table 1).

Lifetime engagement was generally lower among those with diabetes, though the difference for any activity was < 1%. Hypertension followed a similar pattern, except lifetime engagement in any of the activities was much higher for those without hypertension than those with it. Major depression did not follow the same pattern. Lifetime engagement in Hula and paddling was higher among those who had had a diagnosis of major depression in the preceding five years; it was slightly higher for engagement in any of the four activities (Table 1).

Among those with mobility difficulties (difficulty climbing stairs or walking), engagement was higher for Hula and spearfishing, but lower for surfing. Paddling engagement was higher among those without mobility limitations. Overall, engagement in any CRRPA was higher among those *with* mobility limitations compared to those without (Table 1).

**Figure 1:**
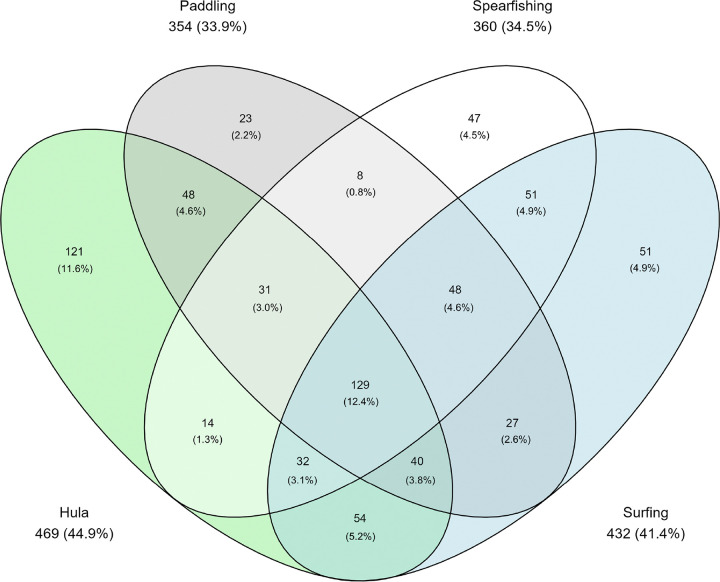
Overlap in reports of lifetime engagement of the four CRRPA activities

Figure 1 presents overlap across the four activities and demonstrates that many respondents participated in multiple CRRPA. In fact, 12.4% of the total sample reported engaging in all four activities at some point in their lifetime. Exclusive engagement in paddling, spearfishing, or surfing was very low (< 5%), while Hula had higher exclusive participation (11.6%).

### Context of Activity

Table 2 presents the context of engagement over the lifetime. Respondents could report more than one context in which they engaged in the CRRPA. Among respondents reporting engagement in Hula, they most commonly did so in a hālau Hula (50.5%), followed by at school (41.8%), with family (32.8%), and/or with friends (27.3%). Outrigger canoe paddling was most common with friends (48.9%), followed by in a paddling club (45.8%), with family (39.6%), and/or at school (22.3%). Among those reporting engagement with spearfishing, it was most commonly done with family (65.3%) and friends (63.3%), followed by alone (15.6%). Almost no one engaged in spearfishing as part of a club or through their church. Finally, for those who engaged in surfing, the most common context of engagement was with friends (77.3%), followed by family (61.1%), and alone (27.6%).

Supplemental table 1 shows the contexts of engagement for those who self-identified as NH. For Hula, the order of the context of engagement was the same as for the full sample; that is hālau Hula, followed by school, family, and friends. For paddling, among NH, engagement in a Club was the most common context of engagement, followed by friends, family and school. For spearfishing, the pattern was the same as for the full sample, as was the case for surfing. For all activities, except paddling, engagement with family members was much higher among NH than the full sample. The differences were + 15.6% for lifetime Hula engagement, + 8.9% for spearfishing, and + 8.2% for surfing among NH compared to the full sample.

### Frequency

Table 3 presents monthly frequency of engagement among those engaged in CRRPA Most participants reported engagement a few times a month, except for Hula, where slightly more reported participation a few times a week. NH participants reported higher daily or near daily engagement for all four CRRPA. For example, 17.4% of NH reported daily or nearly daily engagement in paddling as compared to 14.3% in the full sample. For all activities except surfing, the percentage of NH engaging a few times a week was also higher than the percentage for the full sample (supplemental table 2). These values reflect typical engagement when participants were active, not necessarily current engagement.

### Duration

Table 4 presents the average minutes of engagement for each CRRPA on a day when it was typically practiced. For all activities, mean duration of participation was two hours or more, longest for surfing and shortest for Hula. When compared to the full sample, NH engaged in all CRRPAs for longer periods of time, but the differences between the full sample and the NH sub-sample were quite small.

### Age of Participation over Lifetime

**Figure 2:**
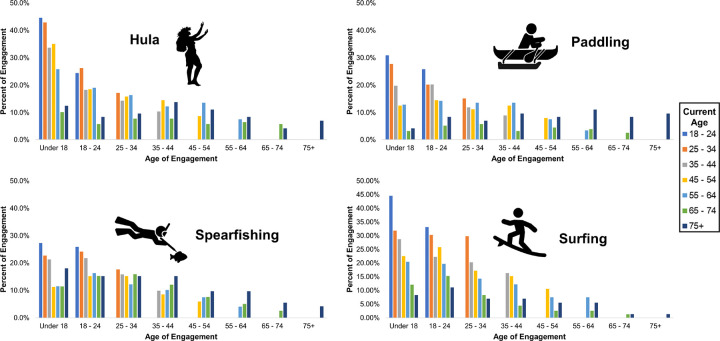
Self-reported ages of CRRPA engagement across the lifespan, by respondent’s age when taking the survey

Figure 2 shows the proportion of respondents that engaged in each CRRPA at different life-phases from early to older adulthood. For each age group on the x-axis, the respondent’s age at the time of the survey is indicated. Because everyone in the survey is 18 years and older, all ages are represented for the age group “under 18”; however, only those 75 years of age and older are represented for the age group “75+”.

Generally, engagement was highest during earlier periods of life and tended to decline with increasing age, but trends varied by current age group and activity. For example, with Hula, a quarter or more of respondents ages 18–64 reported engagement in their youth (< 18). In the younger cohorts, participation during youth surpassed 40%. While engagement dropped off notably after age 18, still, 18.3–26.3% of respondents reported engaging in Hula as young adults (18–24). For older adults in the sample (65 and older), Hula engagement was considerably lower in their youth as compared to the younger cohorts and stayed relatively stable across the lifecourse for those 65–74, while for those 75 and older, engagement was higher when they were in middle-age than when younger. The pattern for spearfishing was different compared to the other CRRPA; across all age groups, the proportion of respondents reporting engagement stayed relatively stable across the lifecourse until around age 45 when engagement started to decline for the older cohorts. Across all activities, the younger cohorts, especially those 18–34, reported greater engagement in youth and early adulthood than the older cohorts.

**Figure 3:**
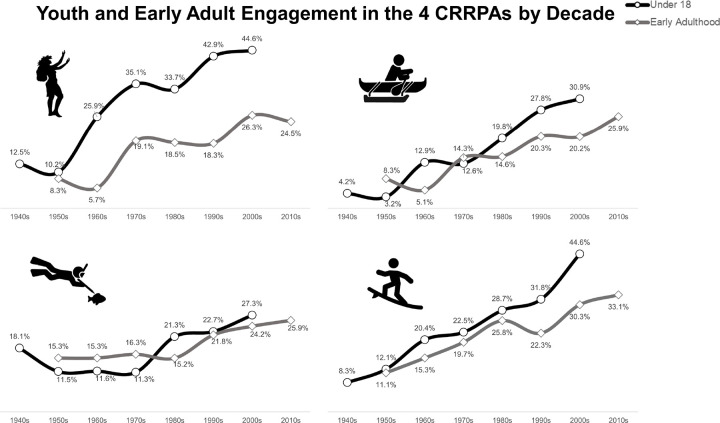
Self-reported youth and early adult engagement in each CRRPA by decade

Because information on youth and early adult engagement in all four CRRPA is available for all cohorts in the sample, trends in engagement were examined across time (Fig. 3). For all of the CRRPA, higher proportions of retrospectively reported youth and young adult engagement appeared in more recent periods, based on the respondent’s current age. For example, for Hula, youth engagement increased by 32.1% between the 1940s and 2000s. For surfing the increase was 36.3%, and for paddling, it was 26.7%. The increase was much smaller for spearfishing (9.2%). Similar patterns were observed for young adult participation. For both Hula and surfing, young adult participation was low across the decades compared to youth participation. For paddling, this was generally but not always true. For spearfishing, young adult participation was higher than youth participation until the 1980s when youth participation surpassed young adult participation. However, the differences between these groups are small (~ 5%) across the lifecourse.

## Discussion

Throughout Hawaiʻi, Hula, outrigger canoe paddling, spearfishing and surfing are popular cultural practices that, for many, remain accessible across the lifecourse, including for those with chronic conditions and mobility limitations. Nearly 70% of the respondents in this survey participated in at least one of the CRRPAs and many participated in more than one during their lifetime. Hula was the most engaged in CRPPA, followed by surfing and then by paddling and spearfishing.

This study was designed to characterize engagement across the state in an effort to draw attention and support health promotion interventions generally and for Native Hawaiians specifically. In doing so, it provides benchmarks and insights for consideration for other CRRPAs in other locations. Lifetime engagement did vary by some sociodemographic and health factors, but for some, like sex, the findings from this survey contradicted much of the literature. This may speak to the relevance of CRRPA to address and promote health equity, and highlight some areas in which the current health promotion scholarship, by undervaluing or undercounting these practices, may have missed critical strengths-based activities that are important to communities.

Most literature globally reports that women engage in PA less than men (C. M. A. Davis et al., 2020; Ominyi et al., 2024; Strain et al., 2024; The Lancet Public Health, 2019). In this survey, there was very little difference in the proportion of men and women reporting lifetime engagement in the four CRRPA activities and overall, engagement was high at around 70% for both sexes. The activities men versus women engaged most in over their lifetimes differed; spearfishing and surfing were more common among men, Hula more common among women, and paddling similarly common for both sexes. When compared with previous work using specially-added questions to the Hawaiʻi Behavioral Risk Factor Surveillance System (BRFSS), for the three activities in common across the surveys (Hula, paddling, spearfishing), there was concordance in the ordering of percent engagement by sex (Hansen et al., 2025; Sentell, 2023). While the similarly high levels of CRRPA by both sexes may reflect the unique context of Hawaiʻi, the results may also suggest that the types of PA assessed in many surveys do not fully capture those in which women engage, leading to underestimates of engagement and the obscuring of promising activities for PA promotion. A similar measurement question was previously raised when quantifying Hula, paddling, and spearfishing engagement among NH and PI in Hawaiʻi (Hansen et al., 2025; Sentell, 2023). As in this paper, previous research on Hula, paddling, and spearfishing using the Hawaiʻi BRFSS reported high levels of lifetime participation by NH (Hansen et al., 2025; Sentell, 2023), while other studies that used standardized questionnaires that omit or discount CRRPA, document low levels of PA in NH when compared to other race/ethnicity groups (Centers for Disease Control and Prevention, 2004; J. Davis et al., 2024).

In contrast to much of the literature on PA, proportionally more lifetime CRRPA engagement was reported among those with lower educational levels than those with higher levels (C. M. A. Davis et al., 2020; Xu et al., 2023). The most notable educational gradient was for spearfishing, which in addition to PA is also a food acquisition activity. A similar result has been reported previously using Hawaiʻi BRFSS data (Hansen et al., 2025). The inverse associations with education may reflect differences in motivations for, preferences, and access to different types of PA by educational attainment.

Consistent with hypertension and self-rated health, respondents reported lower levels of each individual activity if they had diabetes compared to those who did not. However, there was almost no difference by diabetes status in engagement in any of the activities during the lifetime. Thus, those who at the time of the survey had diabetes appear likely to have engaged in multiple CRRPA over their lifetimes. This may reflect higher engagement by NH in multiple CRRPA, as diabetes is higher among NH than other race/ethnicity groups in Hawaiʻi (Kirtland et al., 2015; Mau et al., 2009; Uchima et al., 2019). These results support a focus on what people can do, and might like to do, in health promotion generally, and chronic disease prevention and management specifically.

Lifetime Hula and paddling engagement was proportionally higher for those reporting major depression. Individuals with depression may seek activities that support their mental health. Those engaging in Hula in Hawaiʻi have reported emotional and spiritual benefits (Look et al., 2012; Maskarinec et al., 2015) and overall, dance interventions have been shown to reduce depression (Prudente et al., 2024). There was also relatively high reporting of lifetime engagement in the four CRRPA among those with mobility disability, especially for Hula and spearfishing. For spearfishing, this has been observed previously and may reflect the accessibility of the activity for those who can swim and access shorelines, even if they experience challenges walking on land (Hansen et al., 2025). In Hawaiʻi, Hula has been rigorously studied as an intervention to reduce cardiovascular disease risk including among those who have experienced a serious cardiovascular event; it has been demonstrated safe and accessible (Kaholokula et al., 2017, 2021; Maskarinec et al., 2015). Several of the practices studied here can, and have been, adapted specifically to be inclusive to those with mobility or health challenges, such as AccesSurf Hawaiʻi (AccesSurf, 2025).

Results from this survey demonstrate that engagement in these CRRPA was rarely limited to a single activity. In fact, with the exception of Hula, it was quite unusual for anyone to engage in only one of the CRRPA over their lifetime. This suggests that engagement in one may encourage engagement in others and/or that an interest in culture and place may encourage people to try multiple CRRPA. Once again, this highlights the importance of measuring CRRPA, as people in Hawaiʻi engage in multiple PA over the lifetime, and this engagement goes largely unmeasured by common surveillance mechanisms, leading to underestimates of PA in the community, especially among NH, women, and those with lower educations.

Paddling, surfing, and spearfishing, which were rarely engaged in solely, are all ocean-based PA. There is increasing attention to “blue spaces”, places like oceans and lakes, and their positive influences on physical, emotional, and spiritual well-being (Olive & Wheaton, 2021). In particular, water-based sports are garnering attention in the blue space literature because of their relationship between PA and wellbeing. As part of this, research on blue spaces emphasizes the attributes of “place-based health promotion”(Olive & Wheaton, 2021), a concept akin to CRRPA. For NH specifically, the ocean is considered essential to their existence and it has long been recognized that NH are masters of the ocean in surfing, swimming, fishing, canoe racing, sailing and long distance navigation (Walker, 2008). Thus, for NH, there is a historical and cultural dimension to ocean-based activities that may make them particularly salient to this community, including knowledge transfer of the ocean and stories of the ocean in families and across generations.

For all four of the CRRPA, engagement was primarily social. Nearly half of those reporting engagement in Hula or paddling did so in a formalized social context (hālau Hula or paddling club). Hula was also commonly practiced in school. Engagement with friends was high for all four of the CRRPA and family engagement was particularly notable for NH. Regular participation in social PA may reduce social isolation and loneliness through opportunities for social connection and support and being with others can be a primary motivator for the activity (A. J. Davis et al., 2021). Engaging in PA with others can increase the enjoyment of the activity, as well as subjective energy when doing the activity (A. J. Davis et al., 2021). Perceptions of social cohesion–similarity to the group, friendship, and bonding–have been associated with greater levels of adherence to the activity (Spink et al., 2014). Thus, the social aspects of the CRRPA studied here may have significant health promotion relevance in that they may motivate and sustain long-term PA engagement.

While engagement in all CRRPA was common in Hawaiʻi, when compared with the results for the full sample, both the frequency and duration of engagement was higher for NH. While these results do not reflect the intensity of engagement, such as the metabolic equivalents, they do suggest that these activities are ones that people like to engage in regularly and for long periods of time, especially NH. Notably, for all four CRRPA, average engagement was typically two hours or more. Paddling, spearfishing, and surfing are all nature-immersive activities that people engage in for several hours at a time. These are thus important practices for PA promotion to achieve meaningful PA goals and benchmarks.

Results from this study illuminate trajectories in lifetime participation in four CRRPA for the state of Hawaiʻi. Except for spearfishing, engagement across activities was generally high in youth and then dropped after young adulthood. This pattern is consistent with research on PA generally, but indicates an important challenge for the promotion of PA across the lifecourse. Working age adults, who often have children, may lack the time, money, social support, and childcare necessary to maintain regular PA (Bellows-Riecken & Rhodes, 2008), requiring health promotion interventions, including those encouraging CRRPA, to take into account and address competing priorities and obligations. For example, parenthood is associated with physical inactivity (Bellows-Riecken & Rhodes, 2008), with the effects particularly pronounced among working women (Bellows-Riecken & Rhodes, 2008; Pereira et al., 2007). Previous research has highlighted that social support and childcare are critical to alleviating PA barriers; all of the CRRPA studied here are primarily practiced socially and for NH, very frequently as a family. Adaptations to integrate opportunities for childcare into these popular activities could encourage higher participation during middle adulthood. Interestingly, for spearfishing, across age-cohorts, engagement dropped minimally with increasing age. This may reflect the food acquisition element of the activity, which brings additional benefits to the individual, family, and community beyond PA. It may also reflect that this activity is primarily engaged in by men (Hansen et al., 2025), for whom childcare barriers to PA may be less than women as a result of gender norms (Bellows-Riecken & Rhodes, 2008).

When looking retrospectively, the results for Hula are particularly interesting as there was a large increase in engagement after the 1970s. A Hawaiian cultural reawakening, which started in the 1970s, led to a revival of interest in Hawaiian language, music, outrigger canoe racing, traditional navigation and voyaging, and Hula (Kanahele, 1982). The increase in surfing engagement over time likely reflects a combination of its cultural importance to Hawaiʻi, the “California beachboy culture” of the 1960s, as well as concerted efforts by the International Surfing Association that date back to the mid-1990s to develop the sport globally as part of efforts to include surfing in the Olympic games (isasurf, n.d.).

Insights from this research can be applied to health promotion interventions. It is recognized that successful PA promotion needs to be sustainable, in that people persist in their engagement over time. PA that incorporates novelty may stimulate both interest and enjoyment and promote long-term participation (Lakicevic et al., 2020). Novelty and variation in PA have been highlighted as important for novel skill acquisition and mastery; development of new skillsets that enable expansion into other PA options; improvements in body composition and fitness among both children and adults; and, intrinsic motivation for lifelong PA among youth (Lakicevic et al., 2020). Novelty and variation is inherent to all four of the CRRPA analyzed in this study. For example, Hula has been described as a living art, with different dances accompanying different chants and a centuries long history of stylistic adaptation over time and place (Nakamura, 2025). Spearfishing, paddling, and surfing are all outdoor, water-based activities that require keen attention to the environment including water and wind conditions, and in the case of spearfishing, marine animal numbers, diversity, and behavior. There may also be health benefits associated with changing environmental conditions, as has been documented for surfing as related to the challenging nature of the activity: different coasts, winds, currents and seasons requiring constant adaptation (Britton & Foley, 2021).

Aside from novelty, the literature generally supports the long-term effectiveness of culturally-rooted or culturally-embedded PA interventions. For example, a systematic review of research on the long-term effectiveness of PA interventions among adults from diverse income contexts emphasized the potential of culturally-tailored PA strategies, such as Baduanjin, Tai Chi, and Yijinjing in China (Ominyi et al., 2024). Further, the review highlighted the success of emphasizing the role of culturally-rooted practices in sustaining PA, even in resource-constrained settings (Ominyi et al., 2024). Thus, health promotion PA interventions that build on activities that are culturally rooted and relevant have strong potential for long-term adherence and benefits.

An important conclusion of this study is that common measures of PA used in public health surveillance likely underestimate PA, especially among health disparate groups such as NH and those with lower education. This underestimation has public health implications. Statistics that indicate low PA in certain groups are used to support interventions to increase PA in these groups, based on the assumption that the low PA is a contributor or cause of poor health outcomes. However, if PA is insufficiently estimated, interventions may be misguided and more impactful “causes” of these conditions unaddressed. Further, claims of low PA among certain groups minimizes the activities they are already doing and may contribute to discouragement and marginalization. Better measurement of CRRPA provides opportunities for highlighting which activities are popular and for whom. This information can be leveraged to develop health promotion interventions that build upon these activities in ways that are meaningful to the communities that practice them and that celebrate their cultures, histories, and identities.

This study has many strengths, but also limitations. This is a cross-section study and does not seek to suggest cause and effect. Since it is not longitudinal, prospective changes in activity choices, levels, and duration cannot be assessed. The survey asked respondents to report on lifetime engagement and thus, clearly increases as the age to the respondent increases. This raises the risk of recall bias.

The majority of respondents for this survey came from a research panel and were not randomly sampled. However, those reached by telephone were randomly selected. While sampling was primarily non-random, for indicators that were comparable with research on Hula, paddling, and spearfishing using a large, Hawaiʻi-based, and representative of the state BRFSS sample, results were very similar (Hansen et al., 2025; Sentell, 2023).

This survey categorized engagement into two groups, sometimes/often/very often and rarely/never. This was done in part to compare these results with the two similar studies using the Hawaiʻi BRFSS (Hansen et al., 2025; Sentell, 2023), but also to avoid underestimation of engagement. However, classifying one’s engagement into those categories is subjective and what one person deemed as rarely engaging may have been described as sometimes by another respondent.

This work took place in Hawaiʻi, which has a specific context and history. These activities are practiced in many other contexts, and their health promotion potential may differ by place and population group. This work, however, does demonstrate the strong potential for CRRPA to be applied to health promotion interventions. Population-level information around CRRPA of relevance to other communities should also be considered.

## Conclusions

Engagement in CRRPA is high across the state of Hawaiʻi, demonstrating these activities resonate with the public and especially for NH groups. Trends varied notably by factors relevant for strengths-based health promotion, planning, and policy to achieve health equity. Policy and programs supporting culturally relevant opportunities for PA engagement build from a strengths-based perspective, promoting activities of known value and meaning within communities.

## Supplementary Material

Supplementary Files

This is a list of supplementary files associated with this preprint. Click to download.

• SupplementalTables.docx

## Figures and Tables

**Table 1:** Demographics of Lifetime Participation Overall and by Activity Type (N = 1044; 22 individuals removed from the original sample of 1,066 for no response to the CRRPA items)

		Total Sample	Hula (N = 469)	Paddling (N = 354)	Spearfishing (N = 360)	Surfing (N = 432)	Any CRRPA (N = 724)
		*N (col-%)*	*N (row-%)*	*N (row-%)*	*N (row-%)*	*N (row-%)*	*N (row-%)*
**Sex**	*Female*	670 (64.18%)	372 (55.52%)	231 (34.48%)	182 (27.16%)	246 (36.72%)	470 (70.15%)
	*Male*	374 (35.82%)	97 (25.94%)	123 (32.89%)	178 (47.59%)	186 (49.73%)	254 (67.91%)
**Primary Race/Ethnicity**	*White*	242 (23.18%)	69 (28.51%)	53 (21.90%)	47 (19.42%)	75 (30.99%)	132 (54.55%)
	*Native Hawaiian*	425 (40.71%)	275 (64.71%)	210 (49.41%)	205 (48.24%)	228 (53.65%)	367 (86.35%)
	*Japanese*	142 (13.60%)	43 (30.28%)	22 (15.49%)	40 (28.17%)	42 (29.58%)	81 (57.04%)
	*Filipino*	79 (7.57%)	29 (36.71%)	24 (30.38%)	23 (29.11%)	28 (35.44%)	47 (59.49%)
	*Chinese*	44 (4.21%)	13 (29.55%)	5 (11.36%)	8 (18.18%)	10 (22.73%)	19 (43.18%)
	*Other Pacific Islander*	33 (3.16%)	14 (42.42%)	13 (39.39%)	12 (36.36%)	15 (45.45%)	24 (72.73%)
	*Other Asian*	19 (1.82%)	5 (26.32%)	5 (26.32%)	2 (10.53%)	8 (42.11%)	10 (52.63%)
	*Latino*	25 (2.39%)	8 (32.00%)	11 (44.00%)	7 (28.00%)	9 (36.00%)	17 (68.00%)
	*Other*	35 (3.35%)	13 (37.14%)	11 (31.43%)	16 (45.71%)	17 (48.57%)	27 (77.14%)
**Education Level**	*College Graduate*	413 (39.56%)	150 (36.32%)	104 (25.18%)	99 (23.97%)	146 (35.35%)	252 (61.02%)
	*Some College*	352 (33.72%)	170 (48.30%)	138 (39.20%)	129 (36.65%)	144 (40.91%)	257 (73.01%)
	*High School Graduate*	244 (23.37%)	130 (53.28%)	99 (40.57%)	113 (46.31%)	121 (49.59%)	186 (76.23%)
	*Less than High School*	35 (3.35%)	19 (54.29%)	13 (37.14%)	19 (54.29%)	21 (60.00%)	29 (82.86%)
**County of Residence**	*Honolulu*	544 (52.11%)	254 (46.69%)	178 (32.72%)	170 (31.25%)	237 (43.57%)	374 (68.75%)
	*Hawaiʻi*	202 (19.35%)	106 (52.48%)	79 (39.11%)	83 (41.09%)	91 (45.05%)	154 (76.24%)
	*Maui*	172 (16.48%)	65 (37.79%)	60 (34.88%)	63 (36.63%)	61 (35.47%)	116 (67.44%)
	*Kauaʻi*	126 (12.07%)	44 (34.92%)	37 (29.37%)	44 (34.92%)	43 (34.13%)	80 (63.49%)
**Household Income**	<$15,000	135 (12.93%)	61 (45.19%)	38 (28.15%)	48 (35.56%)	55 (40.74%)	92 (68.15%)
	$15,000-$24,999	113 (10.82%)	60 (53.10%)	55 (48.67%)	49 (43.36%)	53 (46.90%)	92 (81.42%)
	$25,000-$49,999	215 (20.59%)	112 (52.09%)	77 (35.81%)	66 (30.70%)	82 (38.14%)	150 (69.77%)
	$50,000-$74,999	209 (20.02%)	104 (49.76%)	74 (35.41%)	74 (35.41%)	90 (43.06%)	154 (73.68%)
	$75,000-$124,999	217 (20.79%)	85 (39.17%)	67 (30.88%)	76 (35.02%)	90 (41.47%)	142 (65.44%)
	$125000+	155 (14.85%)	47 (30.32%)	43 (27.74%)	47 (30.32%)	62 (40.00%)	94 (60.65%)
**Diabetes**	*No*	903 (86.49%)	415 (45.96%)	310 (34.33%)	315 (34.88%)	391 (43.30%)	627 (69.44%)
	*Yes*	141 (13.51%)	54 (38.30%)	44 (31.21%)	45 (31.91%)	41 (29.08%)	97 (68.79%)
**Major Depression**	*No*	914 (87.55%)	400 (43.76%)	302 (33.04%)	318 (34.79%)	382 (41.79%)	632 (69.15%)
	*Yes*	130 (12.45%)	69 (53.08%)	52 (40.00%)	42 (32.31%)	50 (38.46%)	92 (70.77%)
**Hypertension**	*No*	851 (81.51%)	398 (46.77%)	314 (36.90%)	312 (36.66%)	376 (44.18%)	608 (71.45%)
	*Yes*	193 (18.49%)	71 (36.79%)	40 (20.73%)	48 (24.87%)	56 (29.02%)	116 (60.10%)
**Self-Reported Health**	*Excellent*	111 (10.67%)	59 (53.15%)	55 (49.55%)	54 (48.65%)	64 (57.66%)	85 (76.58%)
	*Very Good*	296 (28.46%)	126 (42.57%)	117 (39.53%)	102 (34.46%)	127 (42.91%)	200 (67.57%)
	*Good*	383 (36.83%)	158 (41.25%)	110 (28.72%)	119 (31.07%)	143 (37.34%)	255 (66.58%)
	*Fair*	211 (20.29%)	107 (50.71%)	60 (28.44%)	75 (35.55%)	85 (40.28%)	157 (74.41%)
	*Poor*	39 (3.75%)	18 (46.15%)	11 (28.21%)	10 (25.64%)	10 (25.64%)	24 (61.54%)
**Difficulty Climbing**	*None*	714 (68.46%)	309 (43.28%)	250 (35.01%)	244 (34.17%)	302 (42.30%)	485 (67.93%)
	*Any*	329 (31.54%)	160 (48.63%)	104 (31.61%)	116 (35.26%)	130 (39.51%)	239 (72.64%)
**Difficulty Walking**	*None*	820 (78.69%)	358 (43.66%)	278 (33.90%)	269 (32.80%)	345 (42.07%)	556 (67.80%)
	*Any*	222 (21.31%)	111 (50.00%)	76 (34.23%)	90 (40.54%)	87 (39.19%)	167 (75.23%)

**Table 2:** Contexts of engagement across the four culturally and regionally relevant physical activities, among those reporting engagement in those activities. Respondents could select more than one context of engagement.

	Hula	Paddling	Spearfishing	Surfing
**Context**	N,%	N,%	N,%	N,%
**Alone**	NA	NA	56 (15.56%)	119 (27.55%)
**At School**	196 (41.79%)	79 (22.32%)	NA	NA
**With Family**	154 (32.84%)	140 (39.55%)	235 (65.28%)	264 (61.11%)
**With Friends**	128 (27.29%)	173 (48.87%)	228 (63.33%)	334 (77.31%)
**In halau hula** * **; a Club**	237 (50.53%)	162 (45.76%)	7 (1.94%)	18 (4.17%)
**Church**	58 (12.37%)	12( 3.39%)	3 (0.83%)	9 (2.08%)
**Other**	30 (6.40%)	11 (3.11%)	8 (2.22%)	4 (0.93%)

* Hula only

**Table 3:** Frequency of engagement, in a typical month, in each of the culturally and regionally relevant physical activities among those who reported lifetime engagement in one or more of them

	Hula	Paddling	Spearfishing	Surfing
**Less than once a month**	52 (11.21%)	52 (14.86%)	75 (21.25%)	50 (11.68%)
**About once a month**	45 (9.70%)	50 (14.29%)	74 (20.96%)	52 (12.15%)
**A few times a month**	159 (34.27%)	112 (32.00%)	126 (35.69%)	165 (38.55%)
**A few times a week**	164 (35.34%)	86 (24.57%)	61 (17.28%)	121 (28.27%)
**Daily or almost daily**	44 (9.48%)	50 (14.29%)	17 ( 4.82%)	40 (9.35%)

**Table 4:** Mean duration of engagement, in minutes, for each culturally and regionally relevant physical activities, when it was practiced on a typical occasion, for the full sample and for Native Hawaiians only

	Mean minutes (SD) for full sample	Mean minutes (SD) for Native Hawaiians
Hula	134.29 (91.05)	141.98 (91.05)
Paddling	169.32 (104.51)	171.79 (105.94)
Spearfishing	176.15 (120.07)	188.54 (135.99)
Surfing	207.78 (157.78)	226.39 (193.41)

## Data Availability

The data analyzed for this study are available upon request of the corresponding author.

## Abbreviations

BRFSS: Behavioral Risk Factor Surveillance System
CRRPA: Culturally and regionally relevant physical activity
NH: Native Hawaiian
PA: Physical activity
